# A Novel Electronic Frailty Index as a Predictor of Clinical Outcomes After Transcatheter Aortic Valve Implantation

**DOI:** 10.1093/geroni/igab046.2990

**Published:** 2021-12-17

**Authors:** Maria Rangel, Rahul Annabathula, Nicholas Pajewski, Jeff Williamson, David Zhao, Kathryn Callahan

**Affiliations:** 1 Wake Forest School of Medicine, Winston-Salem, North Carolina, United States; 2 Wake Forest Baptist Medical Center, Wake Forest School of Medicine, North Carolina, United States; 3 Wake Forest School of Medicine, Winston-Salem, North Carolina, United States

## Abstract

Transcatheter aortic valve implantation (TAVI) is becoming the preferred therapeutic approach for older adults with severe aortic valve disease. Frailty portends increase mortality and adverse outcomes after TAVI. We sought to evaluate an electronic Frailty Index (eFI) as a predictor for increased healthcare utilization, adverse clinical and functional outcomes. We retrospective studied 302 adults older than 65 years that underwent TAVI at our institution between October 2017 and September 2020. The mean age of the cohort was 79 ±6.94 years old; 43% were female. Frail individuals (eFI >0.20), as compared to Fit (eFI <0.10) and Prefrail (0.10>eFI<0.20), were more likely to have a higher society of thoracic surgeons score and a greater burden of comorbidities. Subjects classified as Prefrail/Frail had longer intensive care unit stay post-TAVI than fit individuals (>24 hours: 17% vs 4%, respectively, p 0.02); and trended toward longer hospitalization time and discharge to a setting different than home. The Prefrail/Frail group also had a higher proportion of subjects with persistent New York Heart Association Class III heart failure symptoms 30 days post-TAVI as compared to Fit (14% vs 2%, p 0.04), however both groups demonstrated significant symptomatic improvement post-procedure. No significant differences in 30 day mortality, major adverse cardiovascular events or readmissions were found. TAVI is an effective treatment with a low incidence of early adverse clinical outcomes in older adults regardless of frailty status; eFI could help in identifying and targeting susceptible adults that may require additional resources to recover post-TAVI.

